# Variable mechanical ventilation

**DOI:** 10.5935/0103-507X.20170012

**Published:** 2017

**Authors:** Paula Caitano Fontela, Renata Bernardy Prestes, Luiz Alberto Forgiarini Jr., Gilberto Friedman

**Affiliations:** 1 Programa de Pós-Graduação em Ciências Pneumológicas, Universidade Federal do Rio Grande do Sul - Porto Alegre (RS), Brasil.; 2 Curso de Mestrado Acadêmico em Biociências e Reabilitação, Centro Universitário Metodista IPA - Porto Alegre (RS), Brasil.; 3 Programa de Pós-Graduação em Biociências e Reabilitação e Reabilitação e Inclusão, Centro Universitário Metodista IPA - Porto Alegre (RS), Brasil.; 4 Programa de Pós-Graduação em Ciências Pneumológicas, Faculdade de Medicina, Universidade Federal do Rio Grande do Sul - Porto Alegre (RS), Brasil.

**Keywords:** Ventilation, artificial/methods, Pulmonary gas exchange/methods, Pulmonary ventilation/physiology, Acute respiratory distress syndrome

## Abstract

**Objective:**

To review the literature on the use of variable mechanical ventilation and the main outcomes of this technique.

**Methods:**

Search, selection, and analysis of all original articles on variable ventilation, without restriction on the period of publication and language, available in the electronic databases LILACS, MEDLINE^®^, and PubMed, by searching the terms "variable ventilation" OR "noisy ventilation" OR "biologically variable ventilation".

**Results:**

A total of 36 studies were selected. Of these, 24 were original studies, including 21 experimental studies and three clinical studies.

**Conclusion:**

Several experimental studies reported the beneficial effects of distinct variable ventilation strategies on lung function using different models of lung injury and healthy lungs. Variable ventilation seems to be a viable strategy for improving gas exchange and respiratory mechanics and preventing lung injury associated with mechanical ventilation. However, further clinical studies are necessary to assess the potential of variable ventilation strategies for the clinical improvement of patients undergoing mechanical ventilation.

## Abstract (pt)

**Objetivo:**

Revisar a literatura em relação à utilização da ventilação variável e aos principais desfechos relacionados à sua utilização.

**Métodos:**

Busca, seleção e análise de todos os artigos originais sobre ventilação variável, sem restrição quanto ao período de publicação e ao idioma, nas bases de dados eletrônicas LILACS, MEDLINE^®^ e PubMed, encontrados por meio de busca pelos termos "*variable ventilation*" OR "*noisy ventilation*" OR "*biologically variable ventilation*".

**Resultados:**

Foram selecionados 36 artigos na busca. Após a análise, 24 artigos eram originais; destes 21 experimentais e 3 clínicos.

**Conclusão:**

Diversos estudos experimentais evidenciaram os efeitos benéficos de variadas estratégias ventilatórias variáveis sobre a função pulmonar em diferentes modelos de lesão pulmonar e em pulmões saudáveis. A ventilação variável parece ser uma estratégia viável para o aprimoramento da troca gasosa e mecânica respiratória, assim como para prevenção de lesão pulmonar associada à ventilação mecânica. Entretanto, estudos clínicos são necessários para investigar o potencial destas estratégias ventilatórias variáveis na melhora clínica dos pacientes submetidos à ventilação mecânica.

## INTRODUCTION

Healthy biological systems can quickly adapt to changing environmental conditions and present intrinsic functional fluctuations within each subsystem, including the cardiovascular^(1)^ and respiratory systems.^(2)^ Respiratory physiology is characterized by intrinsic variability in the respiratory components, including the respiratory rate (RR), tidal volume (TV), respiratory times, and respiratory flow.^(3)^ Moreover, pulmonary insufflation has a non-linear opening characteristic.^(4)^ The typical approach to mechanical ventilation (MV) involving the application of positive pressure and adjustments of fixed parameters on mechanical ventilators distinguishes MV from the physiology of the respiratory system.

However, in pathological biological systems, the intrinsic functional fluctuation (variation) is usually lower. The decrease in the variability of RR and TV in patients with chronic obstructive pulmonary disease^(5)^ and prolonged weaning from MV^(6)^ has been documented. In contrast with other systems, the variability of the respiratory system can be easily affected by efforts to improve its function.^(7)^ In MV, ventilatory parameters are modulated by adjustments to the mechanical ventilator, which can be programmed to provide fluctuating ventilatory parameters to replicate some characteristics of spontaneous ventilation in healthy subjects.

Variable mechanical ventilation (VV) attempts to incorporate the physiological basis of spontaneous ventilation during MV and is defined as a ventilatory mode characterized by the oscillation of one or more respiratory parameters. It aims to mimic the variability observed in physiological ventilation and the natural breathing pattern, which changes from cycle to cycle, as well as other physiological parameters, including heart rate and blood pressure.^(8)^

The concept of VV was proposed by Wolff et al. in 1992.^(7)^ The authors postulated that the cycle-to-cycle variation in the relationship between the inspiratory and expiratory times and the level of positive-end expiratory pressure (PEEP) resulted in continuous lung recruitment, thus improving respiratory compliance and gas exchange compared with conventional mechanical ventilation (CV).

Considering that MV is a commonly used intervention in intensive care units, interest in strategies that can increase the variability of the respiratory pattern has grown recently. The objective of this study was to perform a descriptive analysis of the literature on VV, its clinical and experimental application, and the main outcomes of this technique.

## METHODS

This literature review involved the search, selection, and analysis of all original articles on VV, without restriction on the period of publication and language, available in the electronic databases LILACS, Medical Literature Analysis and Retrieval System Online (MEDLINE^®^), and PubMed by searching for the terms "variable ventilation" OR "noisy ventilation" OR "biologically variable ventilation".

The inclusion criteria were experimental and clinical studies that evaluated the use of VV strategies. The exclusion criteria were letters to the editor, brief communications, case reports, historical articles, editorials, commentaries, study protocols, literature reviews, pilot studies, studies using artificial models, and studies not related to the use of VV strategies.

The databases were accessed by three of the four authors at different times, and the articles related to the research topic were selected based on the information contained in the title and abstract. The studies that each researcher selected were shared with the other researchers for confirmation. After that, the selected articles were read in full, and their references were searched to identify other studies that could meet the inclusion criteria and that might not have been identified in the initial search.

## RESULTS

**Figure 1:**
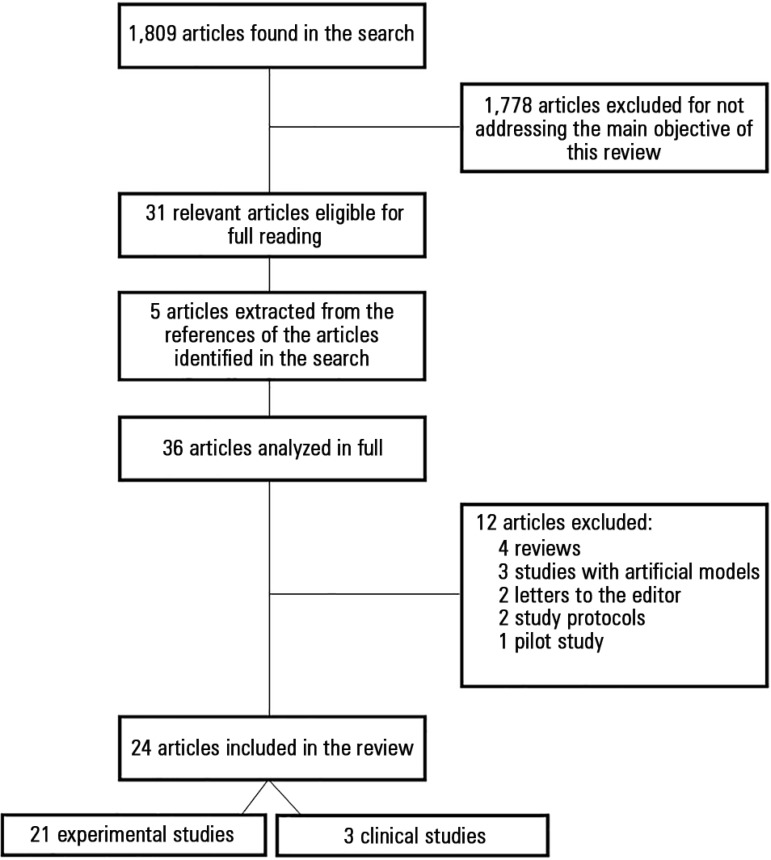
Flowchart of the selection of the studies included in the review.

A total of 1,809 articles were found after searching the selected databases. Of these, 1,778 were excluded after reading the title and abstract because they did not address the central theme of the study. There were discrepancies in the number of articles (28, 30^(9,10)^ and 31^(9-11)^) selected by the three examiners. Five other articles were extracted from the references of the articles identified in the electronic search. The analysis of the 36 articles revealed that 24 were original studies; of these, 21 were experimental studies and three were clinical trials. The remaining were review studies (4), studies that used mathematical or computer models (3), letters to the editors (2), study protocols (2), and pilot studies (1) (Figure 1).

Among the experimental studies, the animal models used were pigs, sheep, and mice, the sample sizes varied between 10 and 64 animals, and the study groups were subjected to different CV and VV strategies. The selected items are shown in table 1.

**Table 1:** Main characteristics of the experimental studies that evaluated variable mechanical ventilation

Author	Sample (N)	Sample characteristics	Objective	Intervention	Conclusion
Thammanomai et al.^(9)^	G1, G2, G3, and G4 = 8 animals in each group with ARDS and 8 animals without ARDS	Mice (22 - 26g) with and without ARDS ventilated for 60 minutes	To investigate the physiological effects of VV and test the hypothesis that the beneficial effects of VV are due to the variability in TV considering its distribution and not simply the presence of large ventilation volumes	G1: CV (TV of 8mL/kg; RR of 240rpm; PEEP of 3cmH_2_O) G2: Original VV (variable RR and TV to maintain the V_min_ of CV) G3: CV with sighs (large breaths, two ventilations per minute) G4: New VV (variable RR and TV - minimum, peak, and maximum volumes - to keep the same V_min_)	The new VV and CV with sighs led to stable dynamic equilibrium in alveolar recruitment that significantly outperformed the CV and the original VV. During the new VV, this balance improved pulmonary mechanics
Berry et al.^(10)^	G1 = 6 animals G2 = 8 animals G3 = 8 animals	Premature lambs (3.2 kg) with 129 days of gestation, ventilated for 3 hours	To assess whether VV is effective for achieving permissive hypercapnia without increasing injury markers or pulmonary inflammation compared with CV	G1: CG (without the use of MV) G2: CV (TV to achieve a PaCO_2_ of 40 - 50mmHg) G3: VV (variable TV and RR to maintain the V_min_ of CV)	VV promoted recruitment and increased ventilatory efficiency without increasing pulmonary inflammation or injury
Bellardine et al.^(11)^	G1 = 6 animals G2 = 7 animals	Sheep (59.8 ± 10.5kg) with ARDS ventilated for 4 hours	To compare VV with CV in terms of gas exchange, hemodynamics, and lung mechanics	G1: CV (TV of 10mL/kg; RR of 16bpm; PEEP of 7.5cmH_2_O; FiO_2_ of 1.0) G2: VV (variable RR and TV to maintain the V_min_ of CV; PEEP of 7.5cmH_2_O; FiO_2_ of 1.0)	VV provided continuous improvement in oxygenation and ventilation pressures and overall better pulmonary mechanics while minimizing pulmonary damage
Mutch et al.^(12)^	G1 = 10 animals G2 = 10 animals	Pigs (20 - 30kg) ventilated for 7 hours	To compare gas exchange and respiratory mechanics in CV and VV during prolonged anesthesia	G1: CV (RR of 15rpm; V_min_ adjusted to deliver a TV of approximately 10mL/kg) G2: VV (variable TV and RR to maintain the V_min_ of CV)	Deterioration of gas exchange and respiratory mechanics occurred with CV but not in VV
Mutch et al.^(13)^	G1 = 9 animals G2 = 8 animals	Pigs (20 - 30kg) with ARDS ventilated for 4 hours	To assess whether VV had positive effects when used with PEEP	G1: CV (RR of 15rpm; PEEP of 10cmH_2_O) G2: VV (variable RR with reciprocal changes of TV; PEEP of 10cmH_2_O)	VV with PEEP of 10cmH_2_O improved arterial oxygenation compared with CV with the same PEEP value
Arold et al.^(14)^	G1 = 4 animals G2 = 10 animals	Guinea pigs (500 - 600g) with ARDS ventilated for 3 hours	To test whether the ability of VV to improve oxygenation and pulmonary mechanics depends on the amount of variability added to TV	G1: CV (RR of 60bpm; TV of 5.1mL/kg, PEEP of 3cmH_2_O) G2: VV (different variations of VT - 10%, 20%, 40%, and 60% of the average - adjustment of RR to maintain the V_min_ of CV)	VV was effective in improving lung function and gas exchange in an ARDS model
Boker et al.^(15)^	G1 = 8 animals G2 = 9 animals	Pigs with ARDS mechanically ventilated for 5 hours	To measure changes in PaO_2_, lung compliance, and proinflammatory cytokines in MV with and without biological variability using an ARDSnet protocol^(16)^	G1: CV (RR of 30bpm; TV of 6mL/kg) G2: VV (variable RR and TV in the same average)	The variability added to the ARDSnet protocol improved oxygenation and reduced the shunting fraction, peak airway pressure, and IL-8 concentrations in the tracheal aspirate
Arold et al.^(17)^	G1 = 6 animals G2 = 5 animals G3 = 5 animals	Guinea pigs (500 - 600g) ventilated for 3 hours	To test whether VV promoted the release of surfactant in vivo	G1: CV (RR of 60rpm; TV of 5mL/kg, PEEP of 3cmH_2_O) G2: VV (variable RR and TV to maintain the V_min_ of CV) G3: CG (Without the use of MV)	VV promoted the release of surfactant, reduced lung damage, and improved blood oxygenation
Funk et al.^(18)^	G1 = 8 animals G2 = 8 animals G3 = 8 animals	Pigs (20 - 30kg) with ARDS ventilated for 5 hours	To compare three ventilation strategies in terms of gas exchange, respiratory mechanics, inflammatory levels, and surfactant function	G1: CV (TV of 7mL/kg; RR of 30bpm; PEEP of 10cmH_2_O) G2: CV with ARM (40cmH_2_O for 40 seconds every hour) G3: VV (variable TV; RR of 30bpm; PEEP of 10cmH_2_O)	VV with a human variability file was greater than CV, and CV with ARM was used for the sustained improvement of gas exchange and respiratory mechanics
Mutch et al.^(19)^	10 animals	Pigs (30 - 40kg) initially with healthy lungs and then with ARDS	To test whether the imposition of a variable respiratory signal with the addition of physiological noise affected cardiorespiratory oscillators	The animals were subjected to MV for 4 to 5 minutes for each ventilation mode - CV and VV (variable RR and TV to maintain the V_min_ of CV) - before and after ARDS	The increase of respiratory sinus arrhythmia by VV may be used to improve the recoupling of organic systems
McMullen et al.^(20)^	G1 = 8 animals G2 = 8 animals	Pigs (25 - 30kg) subjected to selective MV in the dependent lung for 90 minutes and for another 60 minutes after the restoration of ventilation in both lungs	To compare VV with CV in terms of gas exchange and pulmonary mechanics during selective ventilation and after ARM and the reestablishment of ventilation in both lungs	G1: CV (TV of 12mL/kg; RR of 20rpm; PEEP of 5cmH_2_O) G2: VV (algorithm of variability of RR and TV to ensure the V_min_ of CV)	In the selective ventilation model, VV improved gas exchange and respiratory mechanics compared with CV. A better static compliance in VV persisted with the restoration of ventilation in both lungs
Mutch et al.^(21)^	G1 = 9 animals G2 = 9 animals	Pigs (25 - 30kg) with bronchospasm ventilated for 4 hours	To compare VV with CV in terms of gas exchange, respiratory mechanics, CO_2_ exhalation, and inflammatory cytokines in the bronchoalveolar lavage fluid	G1: CV (TV of 10mL/kg) G2: VV (variable RR and TV to maintain a constant V_min_)	VV performed better than CV in terms of gas exchange and respiratory mechanics during severe bronchospasm but without significant differences regarding inflammatory cytokines
Spieth et al.^(22)^	G1 = 9 animals G2 = 9 animals G3 = 9 animals G4 = 9 animals	Pigs (23.8 - 37kg) with ARDS ventilated for 6 hours	To determine the impact of VV on pulmonary function and its effect on pulmonary parenchyma compared with conventional protective MV strategies	G1: CV - ARDSnet^(16)^ G2: VV - ARDSnet^(16)^ (variable TV) G3: CV - OLA^(23)^ G4: VV - OLA^(23)^ (variable TV)	The use of variable TV improved respiratory function and reduced histologic damage during MV according to ARDSnet and OLA protocols without increasing pulmonary inflammation and mechanical stress
Spieth et al.^(24)^	G1 = 8 animals G2 = 8 animals G3 = 8 animals	Pigs (27.2 - 37kg) with ARDS ventilated for 6 hours	To test whether PAV and variable PSV improved oxygenation and reduced the lung damage associated with MV compared with PCV and whether variable PSV further improved oxygenation and reduced lung lesions compared with conventional PSV	G1: CV - (PCV; RR to achieve a pH > 7.25; TV of approximately 6mL/kg, PEEP of 8cmH_2_O) G2: CV - (PSV - free RR, TV of approximately 6mL/kg, PEEP of 8cmH_2_O) G3: VV - (variable PSV - support pressure with a variation of 30% to achieve a TV of approximately 6mL/kg)	PSV and variable PSV reduced lung injury and inflammation and improved gas exchange in relation to protective PCV. Variable PSV further improved oxygenation and reduced inspiratory effort with less alveolar edema and inflammatory infiltration compared to conventional PSV
Ruth Graham et al.^(25)^	G1 = 6 animals G2 = 8 animals G3 = 6 animals G4 = 8 animals	Pigs (10 - 15kg) with ARDS ventilated for 4 hours	To test whether aeration, gas exchange, and pulmonary mechanics were improved when administration of the surfactant was combined with VV	G1: CV (RR of 30rpm; TV of 7.5mL/kg, PEEP of 10cmH_2_O) G2: CV with surfactant replacement G3: VV (variable RR and TV) G4: VV with surfactant replacement	Isolated VV was more effective in reestablishing gas exchange and pulmonary mechanics and had a positive effect on lung recruitment
Graham et al.^(26)^	G1 = 8 animals G2 = 8 animals	Pigs (22 - 30kg) with ARDS ventilated for 4 hours	To test whether alveolar recruitment and periodic breathing with low TV, as observed with VV, increased the resolution of edema in ARDS	G1: CV (TV < 7.5mL/kg, PEEP of 10cmH_2_O; fixed V_min_) G2: VV (variable RR with reciprocal changes in TV to maintain a V_min_; PEEP of 10cmH_2_O)	The CT suggested that the beneficial redistribution and enhanced clearance of pulmonary edema contributed to the beneficial effects of VV
Pillow et al.^(27)^	G1 = 7 animals G2 = 9 animals G3 = 9 animals	Premature lambs with 129 days of gestation ventilated for 2 hours	To test whether VV improved arterial oxygenation, ventilatory efficiency, and lung compliance	G1: CV (PRVC - TV of 11mL/kg; RR of 50rpm; maximum peak inspiratory pressure of 40cmH_2_O) G2: VV (variable TV and RR to maintain the V_min_ of CV) G3: CG (without the use of MV)	VV improved lung compliance and ventilatory efficiency compared with CV
Carvalho et al.^(28)^	12 animals	Pigs (33.1 - 46.6Kg) with ARDS ventilated for 1 hour in each mode	To evaluate the effect of PSV and variable PSV compared to PCV in the regional distribution of aeration, reaeration, and current hyperinflation, and the distribution of ventilation and pulmonary blood flow	CV - (PCV - TV ≈ 6mL/kg; RR to maintain pH > 7.3; PEEP of 8cmH_2_O) CV - (PSV - TV ≈ 6mL/kg; free RR; PEEP of 8cmH_2_O) VV - (variable PSV - support pressure with 20% variation to achieve a TV of ≈ 6mL/kg)	PSV and variable PSV improved oxygenation and intrapulmonary shunting compared with PCV. Compared with PSV, variable PSV redistributed the perfusion of caudal to cranial zones, further improving oxygenation
Spieth et al.^(29)^	G1 = 8 animals G2 = 8 animals G3 = 8 animals	Pigs (26.8 - 34.4kg) with ARDS ventilated for 6 hours	To determine the effect of PAV, variable PSV, and conventional PSV on lung function, respiratory pattern, and lung damage	G1: CV - (PAV - assisted flux of 60%; assisted TV adjusted to achieve a target TV of ≈ 6mL/kg) G2: CV - (PSV - support pressure configured to reach a TV of ≈ 6mLkg) G3: VV - (variable PSV - support pressure with a variation of 30% to achieve a TV of approximately 6mL/kg)	PAV and variable PSV increased the variability of TV and improved the oxygenation and venous mixture without affecting the patient-ventilator synchrony or lung injury compared with conventional PSV. PSV and variable PSV reduced the inspiratory effort compared with PAV
Thammanomai et al.^(30)^	G1 = 8 animals G2 = 8 animals G3 = 8 animals G4 = 8 animals	Rats (22 - 26g) with ARDS	To investigate the combined effects of ventilation modes and PEEP on pulmonary mechanics, gas exchange, and lung biology, including surfactant and epithelial cell integrity, at two PEEP levels	G1: CV (TV of 8mL/kg; RR of 240rpm) with PEEP of 3 and 6cmH_2_O. G2: CV with sighs (large ventilations, two every minute) with PEEP of 3 and 6cmH_2_O G3: New VV (variable RR and TV - minimum, peak, and maximum volumes - to maintain the V_min_ of CV) with PEEP of 3 and 6cmH_2_O G4: CG (received only the initial ventilation after lung injury) with PEEP of 3 and 6cmH_2_O	PEEP had a significant effect on the performance of all the ventilation modes. The higher PEEP protected the lung from collapse and reduced tissue heterogeneity. However, the lower PEEP better protected the epithelium and had a positive effect on the surfactant, particularly during VV
Samary et al.^(31)^	G1 = 12 animals G2 = 12 animals	Wistar rats (365 ± 55g) with pulmonary and extrapulmonary ARDS ventilated for 1 hour	To compare VV with CV	G1: CV (VCV - TV 6mL/kg, PEEP of 5cmH_2_O) G2: VV (VCV - variable TV, coefficient of variation of 30%; PEEP of 5cmH_2_O)	VV improved lung function in both groups. However, VV had further beneficial effects on biological markers in pulmonary ARDS than in extrapulmonary ARDS

G - group; ARDS - acute respiratory distress syndrome; VV - variable ventilation; TV - tidal volume; CV - conventional ventilation; RR - respiratory rate; PEEP - positive end-expiratory pressure; V_min_ - volume-minute; CG - control group; MV - mechanical ventilation; PaO_2_ - arterial pressure of oxygen; PaCO_2_ - arterial pressure of carbon dioxide; FiO_2_ - fraction of inhaled oxygen; ARDSnet - acute respiratory distress syndrome network; IL - interleukin; ARM - alveolar recruitment maneuver; OLA - open lung approach; PSV - pressure support ventilation; PCV - pressure-controlled ventilation; CT - computed tomography; PRVC - pressure-regulated volume controlled ventilation; PAV - proportional assist ventilation; VCV - volume-controlled ventilation.

The sample size of the clinical studies ranged from 13 to 162 individuals of both sexes. These studies evaluated different diseases and respiratory conditions and different CV and VV strategies. The selected items are shown in table 2. The main findings of this review pertaining to the method are summarized in table 3.

**Table 2:** Main characteristics of the clinical studies of variable mechanical ventilation

Author	Sample (N)	Sample characteristics	Objective	Intervention	Conclusion
Boker et al.^(32)^	G1 = 21 patients G2 = 20 patients	Patients who underwent elective aneurysmectomy of the abdominal aorta	To compare CV with VV for pulmonary gas exchange, respiratory mechanics, and radiological evidence of atelectasis	G1: CV (TV of 10mL/kg; RR of 10rpm; PEEP of 0cmH_2_O; FiO_2_ of 0.6). G2: VV (mode with a volume divider - changes in RR resulted in reciprocal changes in TV to maintain the V_min_ of CV)	VV significantly improved lung function compared with CV
Spieth et al.^(33)^	13 patients	Patients with acute hypoxemic respiratory failure who underwent ventilation with conventional PSV and variable PSV for 1 hour each, at random	To compare variable PSV with conventional PSV in terms of pulmonary function and improved patient comfort	Conventional PSV - spontaneous RR; support pressure to achieve a TV of ≈ 8mL/kg; PEEP and FiO_2_ in accordance with current therapy. Variable PSV - support pressure with a variation of 30% to achieve a TV of ≈ 8mL/kg	Variable PSV proved to be safe and feasible compared with conventional PSV; it increased the variability of TV and improved patient-ventilator synchrony, but the rate of gas exchange was similar for the two techniques.
Wang et al.^(34)^	G1 = 83 patients G2 = 79 patients	Older patients subjected to elective resection of gastrointestinal tumor via laparotomy lasting more than 2 hours	To compare two protective MV strategies for cognitive dysfunction during the postoperative period in elderly patients 1 week after open abdominal surgery	G1: CV (VCV - TV of 8mL/kg; RR to reach normocapnia; PEEP of 5cmH_2_O; FiO_2_ of 0.35). G2: VV (VCV - TV of ≈ 8mL/kg with random cycle-to-cycle variation of 30%).	VV versus protective CV decreased the incidence of delirium and cognitive dysfunction in the postoperative period by reducing the systemic proinflammatory response

CV - conventional ventilation; VV - variable ventilation; G - group; TV - tidal volume; RR - respiratory rate; PEEP - positive end-expiratory pressure; FiO_2_ - fraction of inspired oxygen; V_min_ - volume-minute; PSV - pressure support ventilation; VCV - volume-controlled ventilation.

**Table 3:** Key messages of this review

Variable mechanical ventilation
**Benefits**
Improved gas exchange (experimental evidence^(9,11-15,17,18,20-22,24,25,28-31)^ and clinical evidence^(32)^)
Improved respiratory mechanics (experimental evidence^(9,11,13-15,18,19,22,24-26,30)^ and clinical evidence^(32)^)
Improved the ventilation-to-perfusion ratio (experimental evidence^(13,15,18,22,25,28,29,35)^)
Released surfactant (experimental evidence^(17)^)
Reduced the inflammatory response (experimental evidence^(15,17,24)^ and clinical evidence^(34)^)
Reduced lung injury (experimental evidence^(11,17,22)^)
Improved patient-ventilator synchrony (clinical evidence^(33)^)
**Knowledge gaps**
Clinical studies that use randomized controlled clinical trials in different clinical settings, including patients with and without acute pulmonary impairment

## DISCUSSION

The use of VV and its main outcomes were reviewed. VV was evaluated in experimental studies, which reported beneficial effects related to improved lung function, gas exchange, and/or respiratory mechanics without injury and/or inflammation in the lung tissue compared with CV. Nevertheless, VV has been little explored in clinical settings, and only three clinical studies were found in the literature. In addition, these studies had distinct objectives and conflicting results regarding gas exchange.

VV methods are beneficial because they use a nonlinear system to mimic the physiological variability of the respiratory system. These methods may increase TV based on the nonlinear opening characteristics of collapsed alveoli^(8)^ and normal alveoli.^(36)^

Two main epiphenomena form the basis for improvements of lung function during VV: the recruitment and stabilization of pulmonary zones, which contribute to gas exchange, and improvement in the corresponding ventilation-perfusion.

The amplification of ventilated lung zones is primarily achieved by the recruitment of previously collapsed alveoli. Suki et al.^(37)^ demonstrated that once the critical opening pressure of collapsed airways/alveoli has been exceeded, all subtended or daughter airways with lower critical opening pressures will be opened like an avalanche. Considering that the critical opening pressure values of the closed airways and the time required to reach these values may differ among pulmonary regions, the addition of MV patterns that produce distinct airway pressures and inspiratory times may be advantageous for maximizing pulmonary recruitment and alveolar stabilization compared with conventional ventilatory patterns.

To stabilize open lung regions and prevent collapse during MV in healthy lungs, the production and release of surfactant is critical.^(38)^ The release of surfactant increases exponentially with the stretch of alveolar type II cells.^(39)^ Therefore, the high TV generated intermittently during VV may increase the alveolar stretch and thus stimulate the release of surfactant from type II alveolar cells. In healthy mice, random variations in TV promote the endogenous release of surfactant - as shown by the increase in the concentration of surfactant-associated phospholipids and the decrease in the concentration of membrane-associated phospholipids - and improve alveolar stability, thus reducing lung damage.^(17)^ In contrast, in a model of acute respiratory distress syndrome (ARDS) caused by oleic acid, the controlled variable MV showed no benefits to the surface tension of the surfactant based on capillary surfactometry of the bronchoalveolar fluid.^(18)^

During VV, increased gas exchange is usually a consequence of an improved ventilation/perfusion ratio, which results in the redistribution of ventilation to perfused areas and the redistribution of the lung blood flow to better ventilated lung zones. In an experimental model of ARDS, the redistribution of the perfusion occurred from dependent to non-dependent lung zones.^(28)^ A study that used a pig model of ARDS^(22)^ analyzed the lung blood flow using fluorescent microspheres and reported that the variability in TV associated with protective MV strategies redistributed the lung blood flow towards the caudal and peripheral zones. In this sense, VV, by reducing the average airway pressure in ventilated areas and recruiting previously collapsed areas, can reduce vascular impedance and hypoxic vasoconstriction, thus contributing to the adequacy of ventilation and perfusion.

It has been observed that during variable assisted MV (variable pressure support ventilation (PSV)), oxygenation increases despite the absence of improved aeration in dependent lung zones. Variable PSV had no effect on the recruitment or redistribution of aeration compared with conventional assisted MV (conventional PSV) in a saline lung lavage model, and it only affected the redistribution of perfusion from dependent to non-dependent lung zones.^(28)^ In contrast, during variable controlled MV in different ARDS models, there was a reduction in pulmonary shunting^(13,15,18,22,25,35)^ with no significant effect on the dead space,^(15,26)^ suggesting that during variable controlled MV, the reduction in pulmonary shunting is more significant than the reduction in the dead space. Similarly, the venous mixture was reduced in variable PSV but not in conventional PSV.^(29)^

Mutch et al.^(19)^ demonstrated that the application of VV before and after lung injury induced by oleic acid increased respiratory sinus arrhythmia with the addition of variability compared with MV with controlled TV applied during the same periods. The loss of respiratory sinus arrhythmia that occurs in pathological conditions is a consequence of the decoupling of important biological variables. Therefore, measures to restore or enhance the coupling of these variables are advantageous because the increase in respiratory sinus arrhythmia is correlated with a reduction in intrapulmonary shunting and less dead space.^(40)^

Variable controlled MV produced better blood oxygenation than conventional controlled MV in 14 of the 17 experimental studies involving ARDS models,^(9,11,13-15,18,22,25,30,31)^ non-ARDS models,^(17)^ prolonged anesthesia,^(12)^ selective ventilation,^(20)^ and bronchospasm.^(21)^ In three studies, including an experimental ARDS model induced by oleic acid^(26)^ and a preterm lamb model,^(10,27)^ the variable controlled MV did not improve arterial oxygenation compared with conventional controlled MV. The improvement in gas exchange was also evidenced during variable PSV compared with conventional PSV in ARDS models.^(24,28,29)^ Nonetheless, in two clinical studies^(32,33)^ that evaluated gas exchange, only the study by Boker et al.^(32)^ in patients subjected to aneurysmectomy of the abdominal aorta showed significant improvement in this outcome during VV compared to the group subjected to CV. In contrast, in the study by Spieth et al.^(33)^ of patients with acute hypoxic respiratory failure, gas exchange was similar for conventional and variable PSV. However, this study was a randomized crossover trial that used each ventilation mode for only 1 hour, which may explain the similar findings.

In several studies that used experimental models of ARDS,^(9,11,13-15,18,19,22,24-26,30)^ respiratory mechanics were positively influenced by VV. There is considerable clinical evidence in ARDS models^(16,41)^ and non ARDS models^(42-44)^ that higher TV and inspiratory pressure can proportionately trigger or worsen ventilation-induced lung injury because the cyclic opening and closing may increase the shear stress and worsen the inflammatory response, triggering or aggravating lung injury. As in VV, higher TVs are generated randomly and intermittently, critical pressures for opening different airways and alveoli are reached, and lung regions are opened. Therefore, it has been demonstrated that, although high continuous pressures may be harmful, high sporadic pressures resulting from the use of a VV mode may not be harmful and may keep the alveoli open and help open collapsed alveoli.^(35,45)^

Experimentally, Boker et al.^(15)^ suggest that VV may be more protective than CV. They noted that the concentration of interleukin-8 (IL-8) in the tracheal aspirate after 5 hours of VV was lower than that after protective conventional MV, although the degree of pulmonary edema was similar for these two techniques. Corroborating this finding, Arold et al.^(17)^ found that after 3 hours of VV in mice without lung injury, the concentration of IL-6 and tumor necrosis alpha factor (TNF-α) decreased in the bronchoalveolar lavage. These authors also observed that the amount of phospholipids in the bronchoalveolar lavage fluid in VV was similar to that of the control group, whereas this amount was significantly lower in CV, suggesting possible protection against lung injury with the use of VV.

In contrast, several groups found no difference in the inflammatory response between VV and CV. In animal models of ARDS,^(18)^ severe bronchospasm^(21)^ and prematurity,^(27)^ the concentrations of IL-6, IL-8, and IL-10, and total protein content in the bronchoalveolar lavage were similar for both, variable and conventional controlled MV. There were no differences in lung injury in the lung tissues of an animal model of ARDS induced by oleic acid.^(18)^ However, in ARDS induced by surfactant depletion, the variable controlled MV reduced alveolar damage, interstitial edema, hemorrhage, and epithelial dysfunction compared with CV.^(22)^ VV improved lung function without causing structural damage to the lungs or increasing the inflammatory response in the experimental models and, in clinical settings, significantly reduced the systemic proinflammatory response compared with conventional controlled MV during the postoperative period of open abdominal surgery.^(34)^ It is evident that even with the use of non-fixed TV and/or pressure during VV, these variables do not cause inflammatory and structural changes. Moreover, the beneficial effects observed with this method are due to this variability.

Most of the studies analyzed in this review used the variability of RR with a corresponding variable TV or vice versa to provide fixed-minute ventilation.^(9-15,17-22,25-27,30-32,34)^ The exceptions were three experimental studies^(24,28,29)^ and the clinical study by Spieth et al.^(33)^ Recently, the variability of PEEP^(46)^ was evaluated preliminarily in a pig model of ARDS by comparing a protective controlled MV strategy with a similar strategy using two PEEP levels. The variation of PEEP improved gas exchange without causing new lung structural and inflammatory changes.

One study compared the respiratory variability in 10 normal subjects (following 1,587 breaths) with the variability randomly generated by a computer system to evaluate the variability rate related to TV and the impact of gas exchange and pulmonary mechanics. The results indicated that the nature of the chosen variability had no effect on pulmonary function. The authors concluded that the percentage of respiratory variability, but not the pattern of variability, were crucial to the success of VV.^(47)^

The studies analyzed in this review suggest that VV is feasible and can be an effective ventilation strategy for improving lung function, particularly in injured lungs, considering that most of the preclinical studies used ARDS models. Clinical support for VV was presented in three clinical studies,^(32-34)^ but these studies had limitations, including the lack of blinding of the investigator and health care staff, the short-term nature of the investigations, the absence of clinically relevant outcomes, and the small sample size. Furthermore, only two clinical studies provided data on hemodynamics^(32,33)^ and sedation,^(33,34)^ and the latter contained information on the type and prevalence of each sedative but no information on the need for sedatives or the doses used. These factors preclude the inclusion of these studies in clinical practice despite the good results found in the studies analyzed in this review.

Although preclinical studies suggest the benefits of VV in injured lungs with large collapsed and recruitable zones, there is no available data on the use of VV in patients with ARDS. Our group has investigated the role of PEEP variation in gas exchange in patients with mild or moderate ARDS (RBR-5bb65v).

Clinical studies of VV in other populations are underway.^(48,49)^ In 2014, a study protocol was published for a randomized clinical trial^(48)^ of patients who underwent open abdominal surgery lasting at least 3 hours. This study used a TV variation of 30%, considering an average volume of 6 mL/kg/predicted weight. The primary endpoint of the study was the forced vital capacity the first day after surgery. Secondary outcomes included new pulmonary function tests; plasma cytokine levels; spatial distribution of ventilation, assessed by electrical impedance tomography; and pulmonary complications in the postoperative period. Another multicenter controlled randomized clinical study evaluated variable PSV in patients with different pathologies in intensive care units to compare the length of weaning from MV using conventional PSV.^(49)^ The results of these studies, which present a more appropriate design and evaluate more consistent outcomes, may provide further evidence supporting the possible inclusion of VV in clinical practice.

## FINAL CONSIDERATIONS

Variable ventilation may be one of the most extensively investigated ventilation strategies in animal models of disease. Experimental studies have shown the beneficial effects of different variable ventilation strategies for improving lung function and reducing damage in mild to moderate lung injury in the short term. Variable ventilation seems to be a viable strategy for improving gas exchange and respiratory mechanics and preventing lung injury associated with mechanical ventilation. However, little evidence is available from comparative clinical studies with appropriate designs, adequate numbers of patients, and relevant clinical outcomes. Therefore, further clinical studies that use variable ventilation are necessary to assess the potential of variable ventilation strategies for improving the clinical outcomes of patients undergoing mechanical ventilation.
